# Delayed Hospital Presentation and Neuroimaging in Non-surgical Spinal Cord Infarction

**DOI:** 10.3389/fneur.2017.00143

**Published:** 2017-04-12

**Authors:** Slaven Pikija, Johannes Sebastian Mutzenbach, Alexander B. Kunz, Raffaele Nardone, Stefan Leis, Ildiko Deak, Mark R. McCoy, Eugen Trinka, Johann Sellner

**Affiliations:** ^1^Department of Neurology, Christian Doppler Medical Center, Paracelsus Medical University, Salzburg, Austria; ^2^Department of Neurology, Franz Tappeiner Hospital, Merano, Italy; ^3^Spinal Cord Injury and Tissue Regeneration Center, Paracelsus Medical University, Salzburg, Austria; ^4^Division of Neuroradiology, Christian Doppler Medical Center, Paracelsus Medical University, Salzburg, Austria; ^5^Department of Neurology, Klinikum rechts der Isar, Technische Universität München, Munich, Germany

**Keywords:** spinal cord, stroke, ischemia, delay, reperfusion, thrombolysis, neuroimaging

## Abstract

**Background:**

Lack of timely recognition and neuroimaging may be a barrier to reperfusion efforts in acute spinal cord infarction.

**Methods:**

We performed a retrospective study of patients diagnosed with acute non-surgical spinal cord infarction at our tertiary academic center from 2001 to 2015. We studied parameters associated with time from symptom onset to initial hospital presentation and magnetic resonance imaging (MRI) of the spinal cord.

**Results:**

We identified 39 patients among whom anterior spinal artery syndrome was the most frequent presentation (87.2%) and atherosclerosis the most common etiology (56.4%). Nearly, half of the patients presented to the emergency department on the same day of symptom onset (48.7%) but only nine (23.1%) within the first 6 h. Average time from symptom onset to spinal cord MRI was 3.2 days. We could not identify clinical, radiological, or outcome patterns associated with early vs. delayed presentation and imaging.

**Discussion:**

Our study found a time lag from symptom onset to hospital presentation and spinal cord MRI in patients with acute spinal cord infarction. These findings point at low clinical suspicion of spinal cord syndromes and limited recognition as a potentially treatable medical emergency.

## Introduction

Acute spinal cord infarction is a rare disorder, which accounts for only 8% of all myelopathies and not more than 1% of all strokes (1). The condition is provoked by acute disruption of the spinal cord blood supply and results in neurological deficits linked to the supplied vascular territory (2). In the majority of reported patient series, 40–60% of patients had initial American Spinal Cord Injury Association (ASIA) Impairment Scale Grades A and B and a similar proportion remains wheelchair dependent on follow-up several years later (3, 4). Further, the most recent long-term follow-up studies reported a 5-year mortality rate of 21–23% (5, 6).

Two major pathophysiological mechanisms of action can be distinguished for spinal cord infarction. Radicular artery territory infarcts are caused by occlusion of the anterior or posterior artery and central and transverse infarcts are the consequence of systemic hypoperfusion. The main causes of spinal cord infarction are aortic interventions and pathologies, which constitute around two-thirds of the cases (4). The non-surgical etiologies are dominated by atherosclerosis, followed by cardiac and arterio-arterial embolism, global hypoxia, infection, hypercoagulable state, and vasculitis (1, 7). In 20–30% of the patients, the etiology remains unclear. The clinical spectrum of spinal cord infarction is broad and ranges from complete or incomplete spinal cord dysfunction with various degrees of sensorimotor and autonomic disturbances (8). In contrast to cerebral ischemic stroke, many patients with spinal cord infarcts suffer from pain, which is commonly located at the level of the spinal cord lesion. Further, symptoms often develop slower than in supratentorial ischemic stroke, sometimes taking up to several hours before reaching the plateau (9).

Stroke is a neurological emergency. The pressure for rapid stroke recognition and presentation to the emergency department (ED) is largely driven by the proven efficacy of systemic thrombolysis for cerebral ischemic stroke. Indeed, tissue plasminogen activator (t-PA) is an effective therapy for ischemic stroke involving both anterior and posterior circulations within the 4.5 h timeframe (10). More recently, a few case reports delivered insights to feasibility and both potential safety and efficacy of t-PA administration in acute spinal cord infarction (11–15). A timely diagnosis is a pre-requisite to allow administration of t-PA more frequently in patients with spinal cord infarction. For cerebral ischemic stroke, numerous studies examined barriers for the time from symptom onset to arrival at the ED, neuroimaging, administration of t-PA, and benefit for the treatment at a specialized stroke unit (16, 17).

Here, we characterized the spectrum of clinical manifestations and radiological presentations of non-surgical spinal cord infarction and studied potential determinants of pre- and in-hospital delays. Spinal cord infarction accounts only for a small group of ischemic strokes and is not widely known for the spectrum of clinical symptoms, potential treatability, and poor prognosis. Thus, we hypothesized that there are barriers for translating the established standards of care for acute cerebral ischemic stroke to spinal cord infarction.

## Subjects and Methods

### Patient Selection

We performed a retrospective study of patients treated at a tertiary care center from January 2001 to July 2015.

Patients with spinal cord infarction were identified from the hospital database by using following search terms (in German): spinal cord ischemia, spinal cord infarction, ischemic myelopathy, and non-compressive myelopathy.

Thirty-nine patients fulfilled the diagnostic criteria that were

(a) acute or subacute (delay from onset to maximum clinical intensity up to 72 h) spinal cord syndrome,
(b) no plausible alternative explanation based on extensive clinical workup,
(c) radiological investigations with magnetic resonance imaging (MRI) of the spinal cord within 10 days from symptom onset.

The excluded patients had extrinsic spinal cord compression, spinal arterio-venous malformation, systemic and demyelinating disease, and paraneoplastic syndromes. One patient with posterior cord infarction was reported in a previous case study (18).

### Clinical and Paraclinical Workup

Patients underwent standard diagnostic pathways for stroke, which included laboratory testing, evaluation of cardiovascular risk factors, and imaging. When in doubt of the diagnosis, a spinal tap was performed and analyzed for opening pressure, cell count, glucose, protein levels, lactate, hem-derived pigments, oligoclonal bands, immune electrophoresis, and the presence of blood–brain barrier dysfunction. The laboratory workup was amended with testing for autoimmune and paraneoplastic diseases, infections such as neurotropic viruses, HIV, Lyme borreliosis, and *Treponema pallidum*. Spinal MRI (Philips MR 1.5 T until 2007 and later Philips 3.0 T) was performed at least once in all patients. The patients were screened for aortic or vertebral artery pathology based on the level of the spinal cord syndrome. Standard transthoracic cardiac sonography was performed in all patients.

### Analysis of Demographics, Etiology, Clinical Features, and Outcome

We studied demographics (age, sex), clinical symptoms at onset (acute pain in the neck, back, shoulder, chest, abdomen or head, or radicular pain), motor deficits at the ED (motor functions of the worst affected extremity, assessed by Medical Research Council scales), sensory level (the most caudal segment of the spinal cord with normal bilateral sensory functions), joint position sense and vibration testing, and urine retention. Further, we explored whether acute respiratory failure developed or not and mechanical ventilator support was required.

When determining the lesion level we used combined clinical and radiological findings. A follow-up scan was performed if the initial one was negative, or when the clinical course required re-evaluation. We reviewed sagittal T1- and T2-weighted images, axial T2-weighted images at the suspected lesion level and diffusion-weighted imaging, if available. According to the clinical features and MRI findings, patients were classified as having anterior spinal artery (ASA), posterior spinal artery, central cord, or transverse cord infarction. We graded the extent of spinal cord injury with the ASIA Impairment Scale, as reported previously (19).

The etiology was based on the criteria and classification proposed by Nedeltchev et al. (9):

(i) atherosclerosis: history or features of vascular disease or presence of >2 vascular risk factors,
(ii) aortic pathologies: aortic aneurysms or dissections,
(iii) degenerative spine disease at the level of the cord ischemia with the potential of radiculomedullary artery compression in patients without vascular risk factors,
(iv) cardiac embolism defined as the presence of previously or newly established atrial fibrillation without concurrent etiologies,
(v) systemic hypotension defined as an episode of mean arterial pressure below 50 mm Hg, and
(vi) idiopathic.

The analysis of vascular risk factors included following parameters:

- hypertension (systolic blood pressure >140 mm Hg, diastolic blood pressure >90 mm Hg or both),
- diabetes mellitus (symptoms of diabetes plus fasting blood glucose >126 mg/dl or current use of hypoglycemic agents),
- hyperlipidemia (cholesterol normal range >200 mg/dl, triglyceride normal range >150 mg/dl, or current use of lipid-lowering drugs),
- atrial fibrillation (ECG or transthoracic echocardiogram),
- previous cerebral infarctions or transient ischemic attacks (TIAs) (clinical history and chart records),
- myocardial infarction (clinical history, chart records, and ECG),
- heart diseases (congestive heart failure, arrhythmia, heart valve disease),
- renal disease,
- family histories of cerebrovascular attacks, and
- cigarette smoking.

Aortic disorders, particularly aortic dissection, aneurysm, or atheroma, were evaluated non-invasively by transthoracic echocardiogram, computed tomography, or MRI. Diagnostic or therapeutic angiograms were evaluated. We assessed outcome according to medical records at discharge. The categories were in-hospital death or death within 3 months, walk without help at discharge, with help of another person, wheelchair dependent, or bedridden.

### Statistical Analysis

We used χ^2^ test, with Yates correction or Fisher’s exact test, to compare qualitative variables and the non-parametric Kruskal–Wallis test to compare quantitative variables. We performed statistics by using STATA SE 13.0 for Windows (StataCorp LP, TX, USA).

## Results

### Demographics

The search for non-surgical cases of spinal cord infarction revealed 39 patients, 20 were men (51.3%). Most patients were admitted within the last few years. While five patients were identified in the period from 2001 to 2005, this number increased to 11 from 2006 to 2010 and eventually 23 cases within the last 4 years.

Median age was 69 years (range 44–90). Vascular risk factors were dominated by arterial hypertension (64.1%), the runner-up was diabetes (30.8%). One-fifth had a history of cerebral TIA or stroke. Atherosclerosis was the presumed etiology in 56.4% of cases. Further demographics, cardiovascular risk factors, distribution of etiologies, and comorbidities are outlined in Table 1.

**Table 1:** **Demographics of 39 patients with non-surgical spinal cord infarction**.

Parameter	
Mean (SD) age (years)	67.8 (12.4)
Women, *N* (%)	19 (48.7)
Vascular risk factors, *N* (%)	
Hypertension	25 (64.1)
Diabetes mellitus	12 (30.8)
Angina pectoris/peripheral arterial occlusive disease	6 (15.4)
History of stroke/transient ischemic attack	8 (20.5)
Hyperlipidemia	10 (25.6)
Possible etiology, *N* (%)	
Atherosclerosis	22 (56.4)
Aortic pathology	4 (10.3)
Degenerative spinal disease	4 (10.3)
Cardiac embolism	2 (5.1)
Systemic hypotension	2 (5.1)
Iatrogenic	2 (5.1)
Unknown	3 (7.7)

### Clinical Findings and Presentation at the ED

Bilateral and unilateral anterior spinal artery syndromes were the most frequent clinical presentations (71.8 and 15.4%, respectively). ASIA B impairment was most frequent in bilateral anterior spinal artery syndromes (38.5%), a specific time course of clinical disturbances could not be identified when compared to the other syndromes.

A sensory level was found most commonly within thoracic dermatomes (46.1%). Pain history was unclear in 4 (10%) and not reported in 14. Pain preceded other symptoms in 17/21 (73.9%) and developed in conjunction with other symptoms in the remaining 4. Pain persisted until transfer to rehabilitation in the majority. The localization of pain was usually in line with the spinal cord lesion on MRI. However, there was a discrepancy for pain and lesion level in six patients. Autonomic dysfunction was rare and reported only in two cases (5.4%) in terms of urinary incontinence. A detailed presentation of clinical findings is shown in Table 2.

**Table 2:** **Clinical syndromes and time to emergency department (ED) presentation and spinal cord magnetic resonance imaging (MRI) of 39 patients with non-surgical spinal cord infarction**.

	American Spinal Cord Injury Association Impairment Scale, *N* (%)				
Parameter	A	B	C	D	Total
Degree of impairment at presentation, *N* (%)	2 (5.1)	10 (25.6)	19 (48.7)	8 (20.5)	39 (100)
Neurological syndrome, *N* (%)					
Anterior spinal artery	2 (5.1)	5 (12.8)	15 (38.5)	6 (15.4)	28 (71.8)
Anterior unilateral	0	2 (5.1)	2 (5.1)	2 (5.1)	6 (15.4)
Posterior unilateral	0	1 (2.6)	1 (2.6)	0	2 (5.1)
Central	0	1 (2.5)	0	0	1 (2.6)
Posterior syndrome	0	1 (2.6)	0	0	1 (2.6)
Complete spinal cord syndrome	0	0	1 (2.6)	0	1 (2.6)
Neurological level, *N* (%)					
Cervical	0	3 (7.7)	3 (7.7)	1 (2.6)	7 (17.9)
Thoracic	1 (2.6)	5 (12.8)	10 (25.6)	2 (5.1)	18 (46.1)
Lumbosacral	0	0	1 (2.6)	0	1 (2.6)
Overlapping	1 (2.6)	2 (5.1)	5 (12.8)	5 (12.8)	13 (33.3)
Pain reported, *N* (%)	1 (2.6)	6 (15.4)	11 (28.2)	4 (10.3)	22 (56.4)
Autonomic disturbance, *N* (%)	0	0	2 (5.4)	0	2 (5.4)
Mean (SD) days to ED presentation	1.5 (2.1)^a^	0.6 (0.5)^a^	2.7 (4.5)	3.1 (5.4)	2.2 (4.1)^a^
Mean (SD) days from symptom onset to first MRI	1.5 (2.1)	5.7 (14.2)	1.7 (2.0)	4.6 (10.1)	3.2 (8.1)
Mean (SD) days from symptom onset to second MRI	–	7.0 (3.6)	10.2 (11.7)	6.5 (3.5)	8.6 (8.9)

*^a^Calculation excluding one outlier*.

One extreme outlier with ASIA Impairment Scale B was excluded for the analysis of time to hospital admission. The average time from symptom onset to ED presentation in 38 patients was 2.2 days (SD 4.1). There was no significant difference for patients with different ASIA Impairment Scales, as shown in Table 2.

We further examined the courses of 13 patients (33.3%), in whom the exact time (in minutes) from symptom onset to ED presentation was documented. Of them, four arrived at the ED later than 6 h after symptom onset. The affected spinal arterial territory (anterior or posterior), presumed etiology, presence of pain, and ASIA scores on admission were not associated with time from symptom onset to ED presentation (Table 5).

Only nine patients were seen in the ED within the “window of opportunity” of less than 6 h (23%). None of the patients received t-PA. In this acute period, MRT was conclusive in only one patient (number 37 in Table 3). This patient had acute onset of pain in both shoulders accompanied with tetraparesis. MRI showed a T2-lesion from C2 to Th2 consistent with central spinal cord infarction. The sensory symptoms were mild and spinal tap revealed high protein values (85 mg/dl).

**Table 3:** **Clinical presentation and short-term outcome of 39 patients with non-surgical spinal cord infarction**.

No.	Age	Gender	Possible causes	Location of pain	Sensory level	American Spinal Cord Injury Association Impairment Scale Grade	Mobility at discharge	In-hospital death
1	87	M	Atherosclerosis	Whole body	Th12	B	Not mobile	
2	58	M	Degenerative spinal disease	Head and neck	C3–Th4	D	–	
3	76	M	Atherosclerosis		Th11	C	Not mobile	
4	84	F	Atherosclerosis		C3–C7	C	Not mobile	
5	68	F	Atherosclerosis	Buttocks both sides	Th12	D	With help	
6	63	M	Atherosclerosis		Th8	D	–	
7	70	M	Atherosclerosis	Upper abdomen	C3	C		Yes
8	72	F	Iatrogen		Th9	B	–	
9	84	M	Cardiac embolism		Th7	B		Yes
10	54	F	Degenerative spinal disease	Neck with shoulders	C6	B	Wheelchair	
11	75	M	Aortic pathology	Chest and back	Th1	C	Wheelchair	
12	52	M	Aortic pathology		Th4	C	With help	
13	45	F	Unknown	Chest spine	C2	C	–	
14	62	M	Degenerative spinal disease	Both scapulas to arms	C5	D	With help	
15	52	F	Atherosclerosis	Left abdominal	Th5	A	–	
16	69	F	Atherosclerosis	Lumbar	Th12	C	–	
17	58	M	Atherosclerosis	Lumbar spine	Th9	B	–	
18	75	F	Aortic pathology		Th11	D	Wheelchair	
19	68	M	Atherosclerosis	Left quadriceps	Th9	D	Wheelchair	
20	70	F	Atherosclerosis		Th12	A	Not mobile	
21	82	F	Systemic hypotension		Th6	C	Wheelchair	
22	77	M	Atherosclerosis	Inguinal	Th7	C	With help	
23	61	M	Atherosclerosis	Between scapula	C4	D	–	
24	85	F	Systemic hypotension		Th12	B		Yes
25	64	F	Degenerative spinal disease	Lumbar spine	Th12	C	Wheelchair	
26	57	M	Atherosclerosis		Th9	C	Wheelchair	
27	90	F	Atherosclerosis		Th12	C	–	
28	64	F	Atherosclerosis	Both legs	L1	B	–	
29	70	M	Unknown	Lumbar spine	Th4	C	–	
30	70	M	Cardiac embolism		Th10	B	Not mobile	
31	45	F	Idiopathic	Both arms	C5	C	Wheelchair	
32	61	F	Atherosclerosis	Neck both shoulders	C3	B	Wheelchair	
33	79	M	Atherosclerosis		Th9	C	–	
34	70	M	Atherosclerosis	Neck both shoulders	C2	C	Wheelchair	
35	84	F	Atherosclerosis		C4	D	Wheelchair	
36	57	F	Atherosclerosis		Th12	C	Wheelchair	
37	84	F	Aortic pathology		C5	B	Alone	Yes^a^
38	59	F	Atherosclerosis	Left leg	Th6	C	–	
39	45	M	Iatrogenic		Th6	C	Wheelchair	

*^a^The patient died in hospital after transfer to internal medicine due to heart failure*.

*F, female; M, male; C, cervical; Th, thoracal; –, unknown*.

### Spinal Cord MRI

All patients were examined with MRI of the spine; exact times were documented in 18 patients. Median time from admission to first MRI 4:59 h (inter-quartile range 2:10–11:20 h).

The entire spine was studied in 75% of the patients, and exclusive examination of the cervical and thoracolumbar cord was performed in 18 and 7%, respectively. Further details are outlined in Tables 2 and 4. MRI lesions were not detected in four patients (10%) even with repeated spinal cord MRI, these were patients 13, 14, 19, and 38. Spinal CT was performed in 17/39 (22.0%) and 10 patients underwent conventional spinal angiography (25.6%). One patient had an occlusion of the Adamkiewicz artery (arteria radicularis magna).

**Table 4:** **Spinal cord MRI findings of 39 patients with non-surgical spinal cord infarction**.

No.	Time from symptom onset to MRI (in days)	MRI level	DWI positive on first MRI	T2 signal positive on first MRI	DWI positive on second MRI	Spinal angiography
1	5	N/A	No	−		
2	0	C3–Th4	No	−		
3	0	N/A	No	−	No	
4	41	C3–C7	Yes	+		
5	0	Th12–L1	No	−	Yes	Negative
6	5	N/A	No	−	No	
7	4	C3–C7; Th1–Th11	No	−	No	
8	1	Th9–conus		−		
9	2	Th7	No	+		
10	1	C6		−		
11	0	Th1/Th4	No	−		
12	6	Th4–Th6	Yes	−		
13	0	C2–Th2	No	−	Yes	Negative
14	2	C5–Th2	No	+		
15	0	Th5–Th8	Yes	−		Negative
16	7	Th12/conus	No	−	Yes	
17	N/A	N/A		+		
18	3	N/A		−		
19	0	Th9/Th12	No	−		
20	0	Th12–L1	No	−		
21		Th6		−		
22	0	Th7–conus	No	−		
23	0	N/A		+		Negative
24	1	Th12	No	−		
25	0	Th12–L1		−		
26	0	Th9/Th10	No	+		
27	1	Thoracic	No	−		
28	0	N/A		−		Arteria radicularis magna occlusion
29	N/A	N/A	No	+		Negative
30	29	Th10/Th11		−		
31	0	C5/C6	No	−		Negative
32	1	C3/C4	Yes	+		Negative
33	1	Th9–L1	No	−		
34	3	C2/C4		−		
35	1	C4–C7	No	+		
36	0	Th12	No	−		Negative
37	0	C5, C6	Yes	−		
38	1	Th6/7	No	–		Negative
39	1	Th6–conus	No	+	Yes	

*N/A, not available; C, cervical; Th, thoracal*.

**Table 5:** **Time from symptom onset to presentation in 13 patients with non-surgical spinal cord infarction**.

	Within 6 h (*N* = 9)	After 6 h (*N* = 4)	χ^2^	*p* Value
Anterior spinal artery territory (*N* = 9)	77.9	50.0	1.000	0.317
Other arterial territories (*N* = 4)	22.2	50.0		
Atherosclerotic etiology (*N* = 7)	55.6	50.0	0.034	0.853
Other etiologies of spinal cord infarction (*N* = 6)	44.4	50.0		
Pain (*N* = 7)	44.4	75.0	1.040	0.308
No pain (*N* = 6)	55.6	25.0		
American Spinal Cord Injury Association (ASIA) Impairment Scale Grades A–C (*N* = 11)	88.9	75.0	0.410	0.522
ASIA Impairment Scale Grade D (*N* = 2)	11.1	25.0		

*Percentages are shown for patients within and beyond 6 h from symptom onset*.

The first T2-weighted spinal cord MRI was diagnostic in 4/39 (10.3%) of patients, the gain was slightly higher with DWI (5/39, 12.8%). A second MRI of the spinal cord was performed in 29 patients (74.4%), and revealed a T2- and DWI lesion in 10/29 (34.4%) and 4/29 (13.8%), respectively. A third MRI was performed in eight (20.5%). Mean time from symptom onset to MRI was 3.2 days (SD 8.1). A trend for an earlier spinal cord MRI was found for patients with pain (1.2 vs. 5.8 days, *p* = 0.087).

### Laboratory Findings

Workup for vasculitis and autoimmune disorders was performed in 21 (53.4%) patients and was negative in all of them. A spinal tap was performed in 10/39 (25.6%). Elevated protein concentration was found in eight (80%) and a pleocytosis was present in one. Fourteen patients were examined with somatosensory evoked potentials (35.9%) and 9/14 (64.2%) had central conduction deficits.

### Outcomes

Four patients died, details of these patients are shown in Table 3. One patient died on day 6, the remaining more than 3 months after admission. One patient was able to walk without help (2.6%) at discharge, 2 with help of another person (12.8%), 15 (38.5%) were discharged in a wheelchair, and 5 (12.8%) were bedridden. For 13 patients (33.3%), the exact discharge status was unknown. ASIA score at symptom onset was not associated with the clinical status at discharge.

## Discussion

This study characterized the clinical and radiological spectrum of non-surgical spinal cord infarction and analyzed potential barriers to diagnosis and treatment. The retrospective chart review identified 39 patients from a tertiary care center over a period of 14 years with a diagnostic peak within the last 4 years. Nearly half of the patients presented on the same day of symptom onset (48.7%) but only nine (23%) within the first 6 h. Further substantial time lags were identified for spinal cord MRI with average time to a first spinal MRI of 3.2 days. Discrimination of vascular and inflammatory diseases turned out to be challenging in some cases, and also required CSF testing and repeated MRI in a few cases. These findings point at low clinical suspicion of spinal cord syndromes and recognition as a potentially treatable medical emergency. Notably, we could not identify demographic, clinical, or radiological features associated with delays in ED presentation and spinal MRI.

Most infarcts present as an anterior cord syndrome, which results from vascular occlusion or hypoperfusion of the ASA territory and its watershed area (20). In our cohort, the bilateral ASA territory was most frequently affected (72%). Previous studies of non-surgical spinal cord infarction with a similar patient number reported a frequency of 51–75% for anterior cord syndrome (8, 21, 22). Lesion localization in our study was thoracic cord in 46% of patients and the distribution varies largely in the literature (39–75%) (9, 22). The common affection of the thoracic cord might be related to insufficient collaterals in this region and thus the thoracic cord spine being sensitive to systemic hypotension (4). The clinical outcome was unfavorable in about 50%. It needs to be acknowledged that our study is skewed toward an elderly population (67.8 years). Further, we only studied short-term outcome and gradual improvement over up to a year can be observed (4, 5, 23).

Cerebral stroke is characterized by a sudden onset of clinical deficits. This contrasts the situation in spinal cord infarction, where symptoms are often preceded or accompanied by acute pain and develop over a longer period of time (21, 24). Indeed, in non-surgical spinal cord infarction the plateau of sensory, motor, and autonomic dysfunction ranges from minutes to more frequently several hours (8, 20). Patients with cerebral infarcts in comparable cohorts tended to be older have more cardiovascular risk factors and more frequently experience a TIA (24–26). Further, while a history of stroke or TIA is frequent in cerebral stroke, we corroborate previous studies that spinal cord TIAs are rare (8, 9, 22). In our series, vascular risk factors were present in a high proportion of patients and atherosclerosis was the most common etiology. Nowadays, patients, caregivers, and health care professionals are aware of clinical symptoms related to cerebral stroke and recognize time as a key issue for treatment. This awareness can be taken as a result of media campaigns and structural adaptations within healthcare systems by defining stroke-like presentations as a medical emergency (27, 28). Our findings of a delayed presentation at the ED suggest that the clinical spectrum of non-surgical spinal cord infarction is less known in the public and potentially among primary care physicians. In our cohort, neither extent of motor disturbances, pain, nor other factors was associated with earlier hospital arrival. Occurrence of pain was frequent prior to the development of neurological deficits. The clinical presentation, however, might be misjudged as radicular nerve compression, cardiac emergency, or aortic pathology. The reported delays are unfortunate, as spinal cord infarction is associated with detrimental functional outcome (29). Further, timely admission to the stroke unit might be associated with better outcome and thrombolytic therapies appear to have a logical role in the management of spinal cord infarction (6, 29). Stroke unit care has been shown to be beneficial regardless of the stroke etiology following acute ischemic and hemorrhagic stroke (30). Beyond the established stroke unit standards of care, initiation of antiplatelet therapy or heparin would be the obvious consequence with the presence of vascular risk factors or potentially progressive etiologies of spinal cord infarction such as vertebral artery dissection (29). In traumatic spinal cord injury, higher blood pressure correlated with better neurological recovery (31). This observation backs the recommendation to raise the mean arterial pressure above 85–95 mm Hg for the initial 5–7 days in traumatic spinal cord injury. In the lack of study evidence and obvious pathophysiological benefits of improved spinal cord perfusion, this concept could be translated to spinal cord infarction (19, 20). Further, a lumbar drain that allows lowering of CSF pressure to 10–15 mm Hg to improve spinal cord perfusion in spinal cord ischemia may be considered (20, 32). The guidelines of the German Neurological Society have listed a lumbar drain for thoracic anterior spinal artery syndromes which occur during aortic or thoracic surgery. By contrast, the German expert panel concluded that there is not sufficient evidence to recommend thrombolysis or endovascular treatment options. Whether antiplatelet therapy leads to hemorrhagic complications in this context is unclear. Freedman and coworkers also suggest the usage of high-dose steroids in non-traumatic spinal cord infarction according to the traumatic spinal cord injury protocol within the first 3 h due to limited side-effects and lack of proven other treatment options (20). Anticonvulsants administered at therapeutic doses for the management of neuropathic pain were shown to enhance motor recovery after acute spinal cord injury (33). Notably, early administration (i.e., within 1 month) for gabapentinoids, such as pregabalin and gabapentin, was integral for enhanced recovery.

We found substantial time barriers to the realization of thrombolysis and admission to the stroke unit. On the one hand, there are difficulties of clinical recognition and unusual disease evolution due to the slow development of the plateau in contrast to cerebral infarction. On the other hand, it is unclear which examinations in addition to the workup for cerebral stroke are required to rule out potential contraindications. Obviously, an MRI of the entire spine with T2, DWI, and post-contrast T1 is required to evaluate other causes of myelopathy including intramedullary and extra- and intradural pathologies (25). However, the detection of a myelopathy on MRI is not specific for spinal cord infarction. Further, it is a key to rule out life-threatening contraindications of t-PA therapy and causes of spinal cord infarction including aortic dissection. The exclusion of metabolic, cardiac, and cerebral pathologies requires additional diagnostic means such as CT scan of the brain, electrocardiogram, and laboratory testing. When resources are available, some authors also suggest spinal CT arteriography to visualize vessel occlusions and proceed with direct intra-arterial thrombolysis (12, 20). We identified significant delays from symptom onset to spinal cord imaging as another major obstacle to initiate systemic thrombolysis. Yet, whether a time window of 4.5 h or even beyond this period can be translated to spinal cord infarction remains unclear (34, 35). The few case reports available seem to confirm the safety profile within the 4.5 h time frame (11–14). Another issue is the fact that a T2/DWI mismatch concept is not established for spinal cord infarction and dynamics of lesion evolution are only scarcely known. T2-weighted imaging in spinal cord infarction may not visualize a lesion within the first 12–15 h (25). Treatment with t-PA in cerebral stroke is based on exclusion of contraindications and is established in the lack of a detectable brain lesion in the acute phase. This line of thought has yet to be extended to spinal cord infarction. Typical for bilateral anterior spinal cord infarction are hyperintense lesions on axial T2-imaging in the region of the anterior horns illustrating the “snake eye” or “owl’s eye” configuration. Lesions and pathologies on MRI, however, are not uniform and include a pencil-like lesion pattern on T2, various degrees of cord swelling, and enhancement on post-contrast T1 (25). Superiority of early lesion detection by DWI has been reported not only in cerebral stroke but also in spinal cord infarction (25, 36, 37). In our series, a lesion regardless of DWI or T2 was present on initial imaging in 15.4% and we corroborated a slightly higher yield with DWI. The exact time course of lesion development remains to be elucidated. Treatment decision for t-PA or should be guided by clinical presence of a spinal cord syndrome, exclusion of contraindication, and adherence to the time window proposed for cerebral stroke.

The retrospective nature of the study, the number of patients with exact time of symptom onset, and scarce documentation of clinical evolution are limitations of the study. Further, this study reports a single and tertiary care center experience and awaits confirmation from other settings and countries.

## Conclusion

We confirm the notion that early hospital presentation and timely neuroimaging of patients with spinal cord infarction is rare. With regard to opening the time window for thrombolysis and stroke unit care for more patients with spinal cord infarction, we could not identify demographic, clinical, or radiological characteristics, which determine time delays for the diagnosis of spinal cord infarction. There is an urgent need to raise the awareness of this rare syndrome in order to augment the rate of treatment interventions directed at reperfusion. Even if thrombolysis cannot be performed due to contraindications or ED presentation beyond the time frame, stroke unit care might positively affect outcome as for cerebral stroke. Double-blinded randomized studies for spinal cord infarction cannot be expected within the next years. Prospective databases of patients treated with t-PA according to a uniform protocol, which adheres to the established standards, might shed further light to the efficacy and safety of this rare but still devastating cause of permanent neurological disability.

## Ethics Statement

Retrospective and non-interventional studies do not require an ethical approval according to national law. The Medical Ethics Committee of the County of Salzburg reviewed the protocol and waived the requirement for additional approval (415-EP/73/539-2015).

## Author Contributions

JS and SP: concept, data acquisition, data analysis, drafting, and revising. JM: data acquisition and revising. AK, RN, and SL: data analysis and revising. ID and MM: data acquisition and data analysis. ET: data analysis, and revising.

## Conflict of Interest Statement

The authors declare that the research was conducted in the absence of any commercial or financial relationships that could be construed as a potential conflict of interest.
